# Caring for the Older Horse: A Conceptual Model of Owner Decision Making

**DOI:** 10.3390/ani11051309

**Published:** 2021-05-02

**Authors:** Rebecca Smith, Gina Pinchbeck, Catherine McGowan, Joanne Ireland, Elizabeth Perkins

**Affiliations:** 1 Institute of Infection, Veterinary and Ecological Sciences, University of Liverpool, Leahurst Campus, Chester High Road, Neston, Cheshire CH64 7TE, UK; ginap@liverpool.ac.uk (G.P.); cmcgowan@liverpool.ac.uk (C.M.); joirel@liverpool.ac.uk (J.I.); 2 Institute of Population Health, University of Liverpool, Waterhouse Building, Block B, Brownlow Street, Liverpool L69 3GL, UK; lizp@liverpool.ac.uk

**Keywords:** human–horse relationship, companion animal welfare, qualitative research, online forum

## Abstract

**Simple Summary:**

Horse owners are key to facilitating the care that their horse receives. This study sought to understand how management and health care decisions are navigated by owners of older horses. Online discussion forum threads were selected to explore everyday conversations between horse owners around issues of concern relevant to older horse care. Analysis identified seven common themes in owner decision making; the human–horse relationship, horse-related responses, integrated geography, purpose, influences on behavioural outcomes, resources and life worth living. The characterisation of each theme was unique for each human and horse; themes could change over time and were dynamically interrelated. A conceptual model was developed to demonstrate how themes can change in meaning and importance, affecting the human–horse relationship and impacting upon choices made for a horse. This model can be used in the development of practical tools to assist those involved in the care of older horses.

**Abstract:**

The number of aged horses in the UK has been growing over recent years, with many horses remaining active and being cared for into old age. However, increasing age is paralleled with a heightened risk of morbidity and mortality; therefore, owners of older horses must manage changes in their horse, making decisions about management and health care provision. In this paper, we discuss data collected from an open-access online discussion forum, where forum users sought advice arising from concerns about their older horse. Qualitative data analysis was performed using grounded theory methods. A conceptual model was developed to demonstrate the multifaceted ways in which ageing affects the human–horse relationship and impacts upon outcomes for the horse. The model reflects the dynamic nature of caring for an older horse to accommodate change over time—outcomes for the horse shift as the context of day-to-day life changes. The model provides novel insight into how decisions around older horse care are made.

## 1. Introduction

Horses in the UK continue to be looked after by owners into old age and demographics show an ageing equine population [1,2]. Surveys have reported that owners make accommodations for horses ≥15 years of age, including change of use and feed practices [3,4]. Although these populations are at increased risk of chronic disease, with increasing age, there were reductions reported in the provision of routine preventive health care measures and veterinary involvement [3,4].

The way in which people construct their own understanding of the ageing horse impacts on their ideas of disease causality, on relationships with health care providers and on outcomes for health. Despite the presence of strong human–horse relationships, owners are not always best placed to recognise clinical signs of disease or behavioural issues in their horse [5,6]. Where veterinary visits to an animal decrease, there are fewer opportunities for an independent professional assessment of the animal and possible implications for seeking advice. With an overall shift towards reliance on the internet, advice can be sought and experiential knowledge can be shared within online support networks, adding to the construction of lay knowledge [7,8,9]. This knowledge is publicly available and widely accessible via online equestrian community web pages.

In human health care, sociological studies have highlighted the multifaceted process in which people recognise signs of disease as illness, and then navigate health care seeking and treatment [10,11]. We know that owners of older horses recognise changes such as increasing grey hairs, stiff joints/lack of joint flexibility, loss of muscle tone and deepening of supraorbital hollows, often attributing these to signs of ageing [3,12]. However, some of these changes can be associated with diseases amenable to veterinary intervention. Belshaw [13] reported that people caring for older dogs with osteoarthritis found it difficult to interpret behavioural changes in the context of old age. For older adults, the attribution of symptoms to ageing can act as a barrier to treatment-seeking [14]. Little is known about what horse owners feel is important when caring for their ageing horse or the way in which this sits with management and health care decisions.

The human–horse relationship is intricately linked to health: knowledge of the individual, as well as the interactions between human and horse, are known to impact on management choices and decision making around serious disease [15,16]. In this study, we sought to explore horse owners’ experiences of caring for an older horse, looking at the changes and challenges they faced in order to understand how lay knowledge was constructed and the ways in which this impacted on opportunities for care.

## 2. Materials and Methods

In this exploratory study, naturalistic data were utilised to gain insight into publicly presented narratives produced by contributors to an online discussion forum. These observational methods are useful when exploring areas with little previous research [17,18]. The advice sought by horse owners as well as the subsequent discussions taking place in this online equestrian community were analysed.

An internet search engine using the term ‘horse discussion forum UK’ generated a number of forum sites. These were assessed for inclusion based upon the following criteria: open-access, UK based, active in the preceding 4 years (2016–2019), discussion relevant to older horse management and care. One forum met the criteria and was searched for threads using terms relevant to the older horse (See Supplementary Material Table S1). Time frames over which discussions took place were assessed based upon available data. The time and date of posting were visible on recent posts, while in older posts, only the dates were visible. 

Data were collected and analysed using a constructivist grounded theory approach as described by Charmaz [19,20,21]. Forum threads were purposively sampled alongside data analysis. This methodology seeks to understand connections between concepts in the data [21]. By adopting this approach, researchers acknowledge their own role in the interpretation of meaning. RS and EP analysed the data together, discussing possible meanings and interpretations of the content of the threads. The conceptual model of owner decision making was produced through the analysis of horse owners’ narratives of their experiences as recorded in the discussion threads.

Detailed analysis of initial thread data produced conceptual labels or codes. The constant comparison of data enabled codes to be created, refined and combined. The relationships between the codes were identified as a part of the analysis. Codes that were connected were then grouped into larger categories with new conceptual labels, and relationships between categories were examined. The smallest units of analysis, the codes, were used to develop the larger categories and themes, and the overarching analytic framework. Diagrams and models were used to organise data and explore relationships. At all stages of the data analysis, as ideas emerged, they were tested against the raw data in the threads. In total, ten discussion threads containing 280 comments were analysed. The volume of data generated within these threads enabled a point to be reached at which no new codes or categories emerged. In qualitative research paradigms, this is often termed saturation [21]. Findings were then discussed with the wider research team to test resonance and usefulness. 

All quotes have been anonymised for presentation. In some cases, quotes were paraphrased whilst retaining their overall meaning to ensure that individuals could not be identified. A forum user refers to an individual starting a discussion thread, and a contributor refers generally to all individuals participating in the discussion.

## 3. Results

Forum users sought advice by posting their identified question, offering varying degrees of background or context to their concern. Responses to the initial forum question sometimes probed for further details, but most offered advice based on individual experiences as contributors saw them relating to the topic under discussion. The timing of forum posting in relation to any advice-seeking from a veterinarian varied, some users described previous interactions whilst others described intentions of seeking veterinary involvement. Examples of topics arising in discussions included: exercise regimes, farriery, health concerns, retirement, surgical procedures and euthanasia. Time frames over which discussion threads took place varied from hours to days. In some forum threads, there was evidence that the person who had posted the issue had acted upon the advice they had received from the discussion forum.

Analysis produced seven common themes in owner decision making; the human–horse relationship, horse-related responses (physical responses and mental responses were grouped as one conceptual theme), integrated geography, purpose, influences on behavioural outcomes, resources and life worth living. The decision making process was conceptualised as a multifaceted and temporal process, which was navigated within the bounds of the human–horse relationship. For each human and horse, the context of decision making was unique, meaning facets within the themes were constructed differently within each relationship and could change over time. A change in one theme could also prompt an owner to reconsider or reassess the meaning and importance of other themes.

### 3.1. Dynamic Conceptual Model

**Figure 1:**
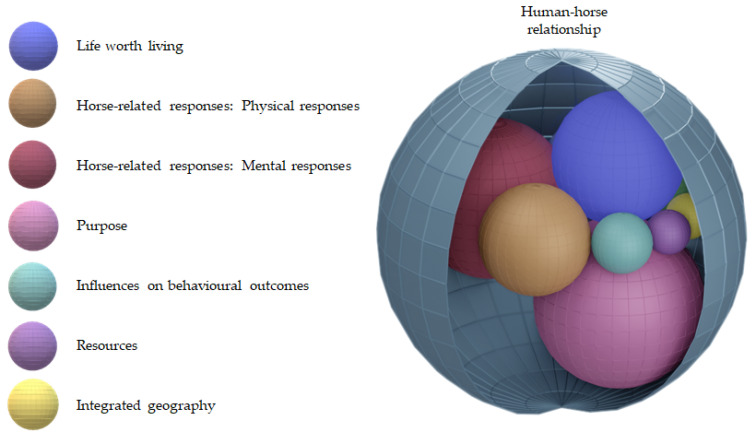
Image of the conceptual model using a theoretical human–horse relationship to represent themes and their interaction at a cross-section in time. The sphere size does not represent quantification as the relative importance of each theme is unique for a particular human and horse at a particular point in time.

Owners talked about the way in which different aspects of the horse’s care became more or less important as the horse aged. These aspects, grouped into the seven themes identified above, were dynamic; interacting with each other and in these interactions producing outcomes, which in turn, had an impact on how the owner managed the horse’s care. A conceptual model was developed to demonstrate the complex interaction between the themes—the six inner themes were subsumed within the overall human–horse relationship. The conceptual model depicted the interacting themes as coloured spheres. When viewed at a cross-section in time (Figure 1), the unique interaction of themes for a particular human and horse are visualised.

The fluidity of life context and its interaction with the human–horse relationship is demonstrated in the conceptual model video (see Supplementary Material Figure S1). As life context for the human and horse altered, themes were at risk of change and the relative importance of a theme (and hence sphere size) also changed at different points in time. The moving spheres have been used to demonstrate this dynamic change. This model enables visualisation of the complexity that is navigated by horse owners, highlighting how priorities around care can shift. The individual themes and their relationships are described in detail below, followed by a case example.

#### 3.1.1. Human–Horse Relationship

The human–horse relationship was the conduit through which care provision was enacted. Although a continuum, the meaning and practicalities of this relationship were constantly reconstructed by the owner as the context of day-to-day life changed.

Contributors understood their horse brought with them a past life, either with the owner or elsewhere, and this became intertwined with the present day. The meaning of this past to the owner impacted upon the characterisation of the horse, their current behaviour or condition, and expectations for the future:

*“I know twenty is not old but she’s an old horse and very high mileage! She’s had a tough life. (Prior to me!)”.*

(Clare)

Through shared experiences between human and horse, older horses were often understood to have earned their care in later life, yet contributors found the navigation of this care challenging. As different themes were weighed up, expectations of the future were drawn upon to navigate what was anticipated to be the most appropriate course of action for the horse. Here the forum user considers the horse’s future lifestyle alongside the recent diagnosis of PPID (pituitary pars intermedia dysfunction, also known as Cushing’s disease or syndrome) and arthritis when making a decision around surgery:

*“He is my horse of a lifetime, done everything for me, but is 20 and recently been diagnosed with PPID and arthritis. He’s already on medication for both, generally doing ok. But is it fair to put him through box rest etc if his future is possibly restricted grazing”.*

(Suzanne)

Knowledge of the individual horse was fundamental to the relationship between the human and horse. This framed the way in which changes in the horse, or life context, were navigated:

*“So i’m pretty certain my lovely twenty three year old boy has had Cushings for a while as he has a few of the symptoms however it hasn’t bothered him. However, over the last week he’s become very sleepy, urination is excessive and just doesn’t look happy! So will be phoning the vet later this morning”.*

(Olivia)

Changes in this horse were understood to have a significant impact on wellbeing, triggering lay advice-seeking prior to veterinary intervention. For others, it was this ability to understand the individual horse within the relationship, which itself was problematic and prompted advice-seeking in the forum.

Contributors often framed their narratives with intentions to provide good care for their horse. They wanted to do the best for them and valued their life. Nevertheless, the construction of optimal outcomes for the horse varied over time and between contributors:

*“I have 2 much loved horses on retirement livery and I rarely see them but get nearly daily photos and videos. I miss them, but know that they’re kept in much better conditions than I can manage … even so, I worry that they feel abandoned!”*

(Grace)

The dynamic conceptual model depicts the interrelatedness of themes within the human–horse relationship. Any of the themes within the horse-human relationship had the potential to change the very nature of the horse-human relationship as constructed by the owner. Section 3.1.8 illustrates the integration of themes with the human–horse relationship.

#### 3.1.2. Horse-Related Responses

Horse-related responses were conceptualised as physical responses and mental responses from the horse, as conceptualised and interpreted by the owner. Although playing separate roles in decision making, these two facets were often talked about in combination. They are represented in the model as two individual spheres that can change independently whilst having a close relationship. They have been grouped together as one conceptual theme—horse-related responses.

The physical body of the horse was often described as if it were an object, over which control could be possible. Boundaries of normal were established over time through knowledge of the individual horse, and contributors also made comparisons between horses to navigate change:

*“She’s holding onto her coat, more so than my medicated mare, drinking more, and although being fed plenty of hay, and shortfeed, still not putting on as much weight as I’d like. If I can’t get a decent weight on her over the summer, she won’t be going through next winter”.*

(Jo)

Mental responses represented the way in which contributors spoke about their understanding of the subjective experience for the horse. Anthropomorphism was used on a number of occasions, and horses were often assigned emotions such as happiness, boredom and contentment. Language such as resilience, tolerance and coping were used to describe the horse and the ways in which the horse responded to, or was anticipated to respond to, life experiences. Here both the horse’s physical and mental responses were reported as important to this contributor when offering advice:

*“If he’s footy on stones and is obviously not happy; then personally I’d shoe him”.*

(Kate)

However, many forum users grappled with the interpretation of the meaning and significance of mental or physical responses that they were noticing in their horse, which prompted advice-seeking:

*“He’s been having seizures, not often but quite bad ones…My problem is that he looks fantastic…So now I’m really struggling with the concept of putting down a horse that looks great”.*

(Louise)

The interpreted meaning of responses could alter with life context. As other aspects of the human’s or horse’s life changed, this could trigger reconsideration of their importance and role in decision making.

#### 3.1.3. Integrated Geography

The theme integrated geography represents the way in which contributors understood themselves and their horse to interact with the built or natural environment. Physical geographical factors included weather and seasonal changes, and the built environment included the yard and its facilities, fields/grazing and stables, for example. The meaning and importance of this theme in decision making could change over time and could also shift along with other themes as the human’s or horse’s needs were understood to change. For example, the suitability of the environment, or the risk it posed, could be reassessed if the horse’s assigned purpose changed.

In the following example, the construction of integrated geography had implications for vaccination protocols. The changing risk of disease resulting from the way in which the horses lived in their environment and activities they were involved in, triggered the forum user to question the need for vaccination:

*“vet mentioned that due to the fact the 2 due are both retired there was little point in continuing....when I enquired about tetanus he said they both had twenty years protection and that that would cover them now…What do you think?”.*

(Billie)

Many considerations were raised by contributors when discussing vaccination in an older horse. Reasons to withhold or cease vaccination included: reduced or more localised activities with the horse, cost, and drug side effects. Additionally, published research regarding vaccination protocols were drawn upon.

Alterations in the environment over the course of a year could pose the human and horse with challenges in daily life and were reported to be important in decision making around euthanasia:

*“I had my 2 old horses put to sleep together, last Autumn… they both had mobility issues and it wouldn’t have been fair to put them through winter”.*

(Kate)

In other contexts, the environment in which the horse was living could be altered or adapted to meet their changing needs:

*“He’s got another few weeks of box rest, but we have plans to section off a 11 × 19 bit of the yard…while our other horse is next to him on the hardstanding turnout”.*

(Suzanne)

Here it was the owner’s understanding of the suitability of the environment, or perceived ability to make changes, which was significant.

#### 3.1.4. Purpose

The theme purpose encompassed two facets: what the horse meant to the owner and outcomes for the horse. The meaning of the horse to the owner relates to what an owner derives from the horse being in their life, such as a horse to ride. Outcomes for the horse were understood to be experiences the horse could access through the opportunities or activities relating to their assigned purpose. In this forum, purpose included being a ridden horse, companion or retiree, and this often changed in later life.

Some contributors wrote of transitions from previous levels of exercise, reducing exercise intensity or frequency in response to changes in their horse. For some, known injury or disease triggered the decision to alter this aspect of management, for others, this was due to a ‘feeling’ that the horse no longer wanted to be ridden:

*“I have been trying to bring her back in to work since the start of July starting with fifteen minutes walk and hacking. She doesn’t feel like there is anything really wrong as such she just feels lacklustre and fed up. I’ve decided to retire her”.*

(Pat)

The construction of outcomes for the horse could shift as their purpose was reassigned. Outcomes such as fitness, weight management or the ability to explore new surroundings, could be replaced by enjoyment with companions at turnout and a degree of increased choice.

Opportunities for health care provision often pivoted around the current or intended purpose of a horse:

*“Boarderline [sic] for me. If the horse was retired and the cost of the operation was to stretch me financially then I would put to sleep. If the horse was happily ridden with a good prognosis to return to that, and if I could afford it, I would be tempted to operate”.*

(Julie)

As with all themes in the conceptual model, purpose interacted with other themes and was not considered alone in decision making.

#### 3.1.5. Influences on Behavioural Outcomes

Contributors described the provision of key components to a horse’s day-to-day life that could be modified to influence the horse’s behavioural outcomes, such as social interactions, nutrition, space, exercise, and pharmaceuticals or nutraceuticals. These were aspects of horse care that could be adjusted or substituted by the owner. Changes in an older horse often triggered contributors to reconsider their provision of these components.

PPID was a common concern in discussions, with forum users recognising changes in their horse, or identifying specific clinical signs and seeking advice around treatment. In other instances, the possibility of PPID was raised by other thread contributors who were suspicious of the disease from the background given in the forum user’s initial post. In these discussions, the provision of the authorised pharmaceutical treatment for PPID, pergolide, enabled the owner to modify their horse’s behaviour:

*“Mine has been on pergolide for six years now and it’s changed him from what I thought was just getting old, to a horse who looks and acts half his age. He did go off his feed in the early days and I just reduced medication for a week and then introduced more slowly”.*

(Marie)

In the sampled narratives, most contributors wanted to be able to medicate and believed this would benefit their horse. For those horses who were on pergolide, dosing was an ongoing challenge in order to achieve what an owner considered to be appropriate behavioural outcomes for their horse.

#### 3.1.6. Resources

Resources identified as significant in the process of decision making were: time, money, support, health care and knowledge. The meaning and weighting of these factors were constructed differently by each contributor and could fluctuate in importance depending on the context. Knowledge relating to the issue of concern, as well as interactions with equine health care providers, impacted on the outcomes for the horse. Care providers mentioned included: veterinary professionals, farriers, complementary therapists and euthanasia service providers (e.g., the hunt).

Individual experiences with care providers were drawn upon by contributors offering advice. It was not only the service they could provide, but the interaction itself that was important:

*“I wouldn’t want a vet to put to sleep my horse if I felt they weren’t supportive though. I’d find someone (another vet or the hunt) who could do it without judgement”.*

(Margaret)

The following contributor describes the decision to employ a different service provider when their own objectives of care were not achieved by a veterinary professional:

*“I wanted it put straight down the vet did not want to (my normal vet was away) I had a blinding flash of light and rang the kennels it was put to sleep one hour later”.*

(Hazel)

This theme became important to decision making when facets were constructed as in increased need, or when limited in availability. These online communities established ideas around the appropriateness of resources to be input to care and could theoretically impact the choices made for a horse by those viewing the discussions.

#### 3.1.7. Life Worth Living

The theme life worth living represented the integration of outcomes for the owner, as well as outcomes for the horse, into an understanding of whether a horse’s life was justified within the relationship. As other themes changed, a contributor’s beliefs around a life worth living were constantly revised.

In the recounting of past decisions around euthanasia, as well as current discussions, outcomes for both the human and the horse were considered:

*“I felt guilty that I could not fix her, but I think I would have felt guiltier if I had stood by and watched her deteriorate”.*

(Fiona)

The meaning and weighing of the outcomes differed depending on life context. For one contributor, their role in the horse’s life was fundamental, and if this was lost, for them, it meant the horse’s life would no longer be worth living:

*“I’m 73 so my health could change in a flash. My farm vet is an all around livestock vet. He did hesitatingly agree he would put to sleep both these senior horses if I get to where I can not care for them”.*

(Alex)

Although in some discussions conflicting opinions were presented, often a social norm was created within the thread itself around what would be an appropriate action. Contributors presented themselves and their actions, justifying past and present decisions and giving advice based on these experiences. These moral judgements were framed as the best outcome for the relationship as a whole.

#### 3.1.8. Case Example

In the following example, the way in which a forum user conceptualised physical and mental responses from their horse had changed due to an unexpected injury. This not only challenged the purpose of the horse, but impacted on the forum user’s construction of the theme influences on behavioural outcomes. In the context of the horse’s new lifestyle the provision of social interactions and exercise were questioned. The forum user narrates their subsequent consideration of relocating the horse to a retirement livery, whilst weighing up financial and time resources required for a management change:

*“I’m worried he is going to get bored out in the field with no purpose. He isn’t in a herd type environment and this was fine whilst he was being ridden as he had a routine of coming in and going out for a ride but to see him sat in the field seems a bit miserable! Any ideas? I’ve thought about retirement livery but I’m not sure how much this works out cost wise……*

*…… The main issue with moving is I have another horse so don’t want 2 in different places as I also work full time so the logistics would be a nightmare. I compete my other horse and the current set up is perfect so can’t move her”.*

(Sam)

**Figure 2:**
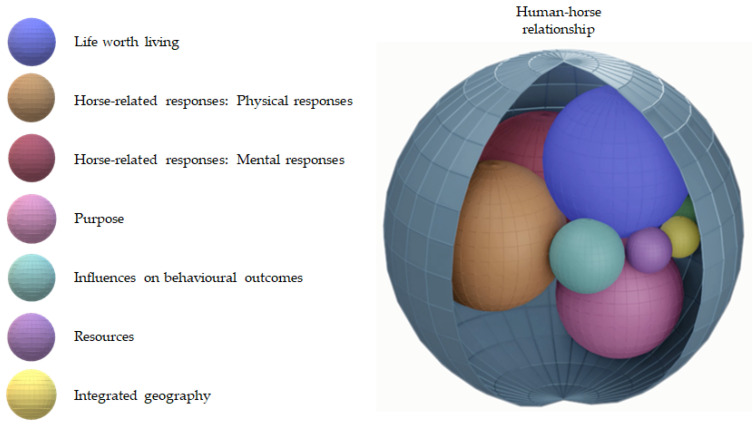
Image of the conceptual model representing the case example human–horse relationship and interaction of themes at a point in time.

This discussion represented a cross-section in time along the life course of the horse at which beliefs around the best way to manage the horse were shifting. Figure 2 shows how the conceptual model can be used to represent the interaction of themes at a point in time.

Advice and reaffirmation about the relocation were sought from those with experiential knowledge in the online community. Over the course of the discussion, knowledge was co-constructed, and the meaning of themes to the forum user was expanded upon. For example, other contributors suggested options available to this owner, including euthanising the horse. The forum user’s response gave insight into their construction of the theme life worth living. The outcomes for the owner from the relationship had changed, but the horse’s life remained justified:

*“I have a horse to ride so wouldn’t put him down because ‘I want a horse to ride’! This horse owes me nothing and I am more than happy to find a suitable solution for him”.*

(Sam)

Here the horse is understood to have earned its care in later life and the human–horse relationship frames the decision around altered care provision. At this time, the meaning of the relationship was being reconstructed in line with the influence of other themes. The resulting consideration of relocating the horse to a third-party care provider shows the dynamic way in which a change in one part of the model can impact on the construction of other themes during the decision making process. This forum user’s difficulty in navigating a change in life context and their subsequent advice seeking highlights the way in which some owners grapple with change. The conceptual model gives insight into the decision making process and how changes interact with the human–horse relationship itself.

## 4. Discussion

This conceptual model developed from forum data gives a novel insight into the dynamic ways in which different factors are weighed up by horse owners within their life context, and the intimate way in which this reconstructs the human–horse relationship. As the context of day-to-day life changes, the meaning and weighting of different facets can alter and impact on the way in which decisions are made for a horse.

The possibility for the human–horse relationship itself to change over time, along with the evolving meaning of other themes in the model, brings a new dimension to the construction of these relationships. As the human–horse relationship changes in meaning, so too can choices around the care and management of the horse. In her work, Shir-Vertesh describes pets as “flexible persons”, where human perceptions of the pet’s role within a family were found to change with life context, in some cases resulting in altered treatment of the pet, or possible relinquishment [22]. This mirrors the findings in our study, where themes were found to have temporal meaning that could develop with changing life context. With this, themes could be reconsidered or reassessed by the human, reconstructing the human–horse relationship and impacting on care and management decisions made for the horse.

The way in which owners conceptualised the horse impacted on the way they understood the importance of change and whether or not action was required. As horse-related responses were considered in the context of other themes, a physical change did not always trigger advice-seeking. Its significance depended partly on the human’s perception of its impact on the human and horse, and their relationship. Discourse around ageing and the expectation of stiffness, for example, can make interpretation of the significance of behaviours challenging for horse owners. This, in turn, is influenced by other constructs such as ideas about a life worth living and the impact a change is understood to have on this. In adults, the severity and rate of symptom occurrence or change, its impact on daily life and on relationships, as well as anticipated relevance of symptoms to a disease process, are known to influence treatment-seeking behaviours [23,24,25,26]. Recognising the dynamic interaction between themes in this model enables understanding of how treatment seeking is navigated through the integration of all parts of the human and horse’s life together. As the construction of themes shift over time, so too can priorities for care, and it is important to recognise this complexity in decision making [27].

Increased awareness of clinical signs of disease, as well as the subtleties associated with pain behaviours in horses, may be needed by those caring for older horses. Dog owners who had been forewarned about the possibility of osteoarthritis in their dog found signs of disease easy to recognise, however for other owners, advice-seeking was prompted by more exaggerated responses such as vocalisation [13]. The ability of a person to detect slow change over time is described as a challenge in quality of life assessment in animals and this is likely to be relevant to issues that develop in older horses [28]. The conceptual model presented in this paper shows that as a horse ages, there are many interrelated changes occurring within the human–horse relationship. Knowledge forms only one facet in decision making and the way knowledge is applied in practice will depend on the construction of other themes in this conceptual model at a point in time.

In the field of animal welfare, a shift towards trying to measure and value the positive experiences and mental states in animal welfare assessments has meant the concept of a life worth living has increased in utility [29,30,31]. Here we add to this concept, using this theme to represent the way in which owners weigh up outcomes for themselves, as well as their horse, when constructing ideas around the meaning of the horse’s life. The concept of life worth living has been described as “an animal life that humans judge to be worth the animal living” [32]. The key is the human assessment of this life, and the conceptual model presented shows that this is not an independent, static judgement. Whether a life is considered worth living shifts with the context of day-to day-life.

In this study, owners often reported their emotional distress associated with different stages of treatment-seeking. Challenges were faced not only in understanding the horse, but in deciding upon acceptable outcomes, including the possibility of the loss of the horse. These emotions are likely to impact on the owner’s own values or priorities in decision making for a horse [28]. Similar emotional distress was reported by owners providing care for chronically ill dogs [33]. Here, owners described experiencing fear of loss of the dog, worry, and difficulty in managing aspects such as welfare, finances and time [33]. Therefore, further research into the best ways to provide support to owners in their journey of caring for an older horse is needed for the benefit of both human and horse.

Analysis of online threads on equestrian community web pages gave insight into how a group with different perspectives on care negotiated their experiences. By coming together to focus on a specific issue of concern, limits to socially acceptable outcomes were established. In some threads, consensus was clear, for example, when debating a decision around euthanasia, the human’s knowledge of the individual and right to end the horse’s life were emphasised in importance. In other threads, the consensus was less clear and debates arose. Birke et al. discuss how social communities can create consensus around ideas of what is good for a horse [34]. In these forum data, the interaction between contributors appeared to partly depend on the primary intentions of participation. Forum users who posed questions often chose to respond to advice that supported their initial preferred course of action. In online communities, contributors can be selective about which entries they engage with and the information they choose to share with others. In these virtual sites, it is perhaps easier to be selective about what advice is acknowledged or acted upon. In addition, the social context in which the human and horse live is also likely to impact on their choices, as well as practical outcomes of care.

In an online survey in which leisure horse owners were asked about where they sought advice on areas relating to their horse, the information source reportedly used varied with the subject of concern [9]. The vet/farrier was selected more frequently than the internet/forums for health advice, however, participants tended to select more sources of advice regarding health than they did for advice on stable care, for example [9]. In this study, information seeking alongside veterinary involvement was documented, which demonstrates the integration of multiple information sources in decision making for an older horse. The construction of the role of veterinary expertise was variable throughout discussions. Experiences with veterinarians were reported, which appeared to have long-term impacts on the interaction with professionals and advice given to others. Some horses may have theoretically benefitted as a result of these online discussions. Forum users posing questions were sometimes informed that observed changes might be indicative of disease and were encouraged to seek veterinary attention. However, the receipt of knowledge in this context could not be relied upon. In a study of Australian pony club members, friends or knowledgeable horse people were primarily consulted about health concerns, with veterinarians often referred to in a negative context and only consulted as a last resort [35]. Therefore, further research into the relationships between horse owners and veterinary professionals is needed to understand how these relationships could be improved.

These findings represent an analysis of data from a subset of the equestrian community. These are horse owners who chose to use the equestrian community web pages to source advice, or share views and advice and so may not be a representative sample of horse owners. However, participation in online communities is not bound geographically and so is likely to encompass a cross-section of the horse-owning population. This study was based on a small sample in which data were purposively selected to include threads relevant to the research focus. This method introduced the possibility of judgement bias as the researcher themselves strategically sourced data to include in analysis and therefore findings may not be generalisable to the wider horse population [36]. Non-participant methods were used, and therefore, only public volunteered data were included. This meant that further detail on the views expressed was not obtained. In addition, the practical outcomes for horse and owner arising subsequent to the discussion were only addressed in some instances and were self-reported. Despite these limitations, understanding of the way in which horse owners seek advice and utilise an online forum for discussion has been discussed. Further research could include interviews with horse owners to enable a more in-depth exploration of their experiences and the accounts they give. This would give an opportunity to focus on areas of interest, as well as giving insight into how online information seeking is situated within the wider decision making process.

## 5. Conclusions

The development of a conceptual model involving seven dynamic themes has provided novel insight into horse owner decision making. The model can be used to understand the way different facets of life interplay, producing altered perspectives around care. This model will be useful for horse owners, as well as health care providers, as a tool to aid reflection and to establish priorities around care at a point in time. By visualising this social process, the model could be used to aid identification of the impact of changes on the life of an older horse, and it can be used during discussions between horse owners and other care providers to aid communication at times of decision making.

## Data Availability

The data analysed and presented in this study are from publicly available datasets. The data are available from the corresponding author upon reasonable request.

## Supplementary Materials

The following are available online at https://www.mdpi.com/article/10.3390/ani11051309/s1, Table S1: Search terms used within forum Figure S1: Video of the conceptual model showing the changing importance and dynamic interaction of themes over time within the human–horse relationship.
