# Supplementary material for: Caring for the Older Horse: A Conceptual Model of Owner Decision Making
Source: Animals (Basel). 2021 May 2;11(5):1309. doi: 10.3390/ani11051309 (PMC8147395; doi:10.3390/ani11051309)
Supplement: Supplementary file 1 [file animals-11-01309-s001.zip › Supplementary Material Table 1.pdf]

Supplementary Material Table 1: Search terms relevant to the older horse used to search for discussion fora threads.

Search terms	old horse, old horse care, geriatric horse, elderly horse, vaccination old horse, vaccinate old horse, PPID, cushing's, vet old horse, quality of life horse, retirement, retirement livery, retirement yard
